# Chemoprophylaxis for the prevention of tuberculosis in kidney transplant recipients: A systematic review and meta-analysis

**DOI:** 10.3389/fphar.2023.1022579

**Published:** 2023-03-16

**Authors:** Zhihui Yuan, Sheng Chao, Yuan Xu, Yulin Niu

**Affiliations:** Department of Transplantation Surgery, The Affiliated Hospital of Guizhou Medical University, Guiyang, Guizhou, China

**Keywords:** isoniazid, prophylaxis, tuberculosis, kidney transplant, meta, analysis

## Abstract

**Background:** A systematic review and meta-analysis was performed to investigate the efficacy and safety of isoniazid (INH) prophylaxis to prevent tuberculosis (TB) infection in kidney transplant recipients (KTRs).

**Methods:** Web of Science, SCOPUS, and PubMed were searched to identify relevant studies that compared the effects among patients who received INH prophylaxis after transplantation.

**Results:** A total of 13 studies (involving 6,547 KTRs) were included in our analysis. We found that the risk of active TB infection (RR: 0.35, 95%CI 0.27–0.45, *p* < 0.01) for KTRs was lower in the INH treatment group than in those without prophylaxis. However, there was no significant difference between the two groups in mortality (RR: 0.93, 95%CI 0.67–1.28, *p* = 0.64), acute rejection (RR: 0.82, 95%CI 0.44–1.51, *p* = 0.52), and hepatotoxicity (RR: 1.25, 95%CI 0.94–1.65, *p* = 0.12).

**Conclusion:** Isoniazid prophylaxis is a safe and effective for KTRs on reactivation of latent TB infection.

## Introduction

Kidney transplantation (KT) is the most important replacement therapy for patients with end-stage kidney disease (ESKD) (Lamb et al., 2011); it can improve the prognosis and life quality of ESKD patients (Park et al., 2020). With the wide use of immunosuppressive drugs, the survival rates of patients and grafts have improved remarkably (Singh et al., 2016; Hosohata et al., 2018), while opportunistic infections caused by excessive immunosuppression have also increased (Fang et al., 2021).

Tuberculosis (TB) has been a serious infectious disease in solid organ transplantation (SOT) (Roth et al., 2016; Clemente et al., 2018; Burguet et al., 2022). It is reported that the incidence of TB in SOT recipients ranges from 0.56% to 2.61% (Reis-Santos et al., 2013), which is 20 to 74 times higher than in the normal population (Subramanian and Dorman, 2009; Epstein and Subramanian, 2018). The mortality rate of TB infection after transplantation is 31% (Horne et al., 2013; Baker et al., 2017; Majeed et al., 2018). Due to atypical clinical symptoms, diagnosis may be delayed in some patients with active TB infection (Yi and Cheng, 2020). In addition, the interaction between anti-TB drugs and immunosuppressants makes diagnosis and treatment more difficult. Most active TB infections are considered to develop from the reactivation of latent tuberculosis infection (LTBI) after transplantation (Abad and Razonable, 2018; Sasi et al., 2020). Therefore, some experts have suggested that a TB prophylaxis strategy should be used in SOT recipients to reduce the incidence of post-transplant active TB infection (Naqvi et al., 2010; Adamu et al., 2014; Dodani et al., 2021). Some transplant centers use isoniazid (INH) prophylaxis in SOT recipients based on clinical experience, but this has been controversial, with side effects such as affected liver functioning and low medication compliance. Although some analogous meta-analyses have all been performed on clinical studies (Currie et al., 2010; Adamu et al., 2014), there is still a lack of comprehensive and accurate meta-analysis of INH prophylaxis among such patients. Thus, we conducted a systematic review and meta-analysis of relevant studies to evaluate the safety and efficacy of the INH prophylaxis strategy in KTRs.

## Materials and methods

Our systematic review was conducted and reported according to the PRISMA (Preferred Reporting Items for Systematic Reviews and Meta-analyses) guidelines (Moher et al., 2009), presented in Supplementary Table S1.

### Search strategy

Web of Science, SCOPUS, and PubMed were searched for all relevant studies up to 10 June 2022 (the latest search date) using logical combinations of relevant keywords: “kidney/renal transplant/allograft, tuberculosis”. The full text of the search terms for each database is presented in Supplementary Table S2. In addition, eligible references from relevant studies were also searched. All articles identified by this search strategy were evaluated by two independent reviewers (ZY and SC) according to their title, abstract, and full text to determine the final included studies. Studies about adult KTRs receiving INH prophylaxis for TB were included. When one study was reported many times, the study with a long follow-up period and complete case report was identified as the primary data source.

### Outcome measures

The primary outcomes in this review were active TB infections after transplantation and all-cause mortality. The secondary outcomes were hepatotoxicity and acute rejection (AR).

### Data extraction

Two investigators (ZY and CS) independently identified eligible studies by assessing the title, abstract, and full text of all studies. Data extraction was then independently performed by two reviewers (ZY and CS) according to the following items: study design, participant characteristics, interventions, and outcomes. Any data discrepancies were resolved by the whole team, and missing information was requested from study authors or sponsors.

### Quality assessment and statistical analyses

We used Cochrane’s risk of bias to assess the methodological quality of all included studies (Higgins et al., 2011). The fixed effect model or random effect model was selected in a meta-analysis, depending on the value of heterogeneity. Heterogeneity across the studies was assessed using Cochrane’s Q (*p* < 0.1) and I^2^ statistics (I^2^ >50%). I^2^ values of 25%, 50%, and 75% corresponded to low, medium, and high levels of heterogeneity, respectively. If there was a value of more than 75% regarding heterogeneity (Higgins et al., 2003), a sensitivity analysis, subgroup analysis, or meta-regression analysis was performed to explore possible sources of heterogeneity. Data were pooled using the mean difference (MD) as the effect estimate, and the binary outcomes were presented as risk ratios (RRs) with 95% confidence intervals (CIs); *p* < 0.05 was considered statistically significant. All analyses were performed in R (version 4.1.1, R Project for Statistical Computing).

## Results

### Characteristics and quality assessments of included studies

**FIGURE 1:**
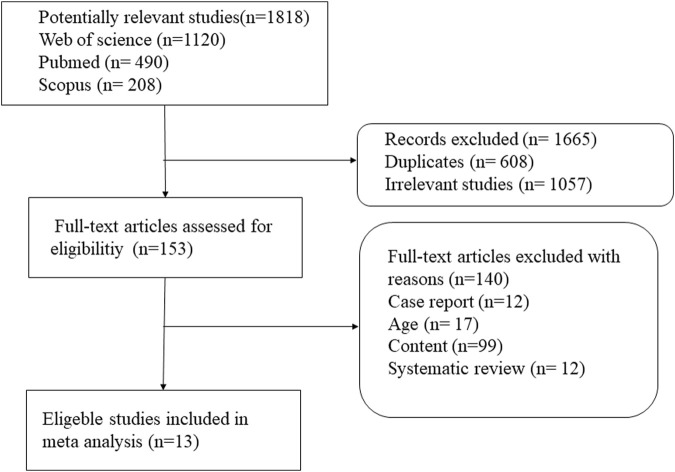
Procedure for search and selection of studies included in the systematic review and meta-analysis.

**FIGURE 2:**
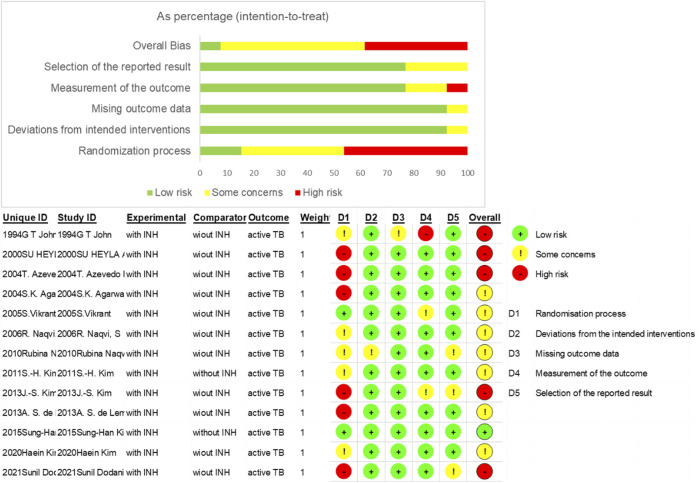
Risk of bias for included studies.

Some 1,818 potentially relevant articles were identified (Figure 1), of which 1,665 duplicates and irrelevant studies were removed. After screening titles and abstracts, 153 full-text articles were assessed. Finally, 13 studies (which included 6,547 patients) met the inclusion criteria (John et al., 1994; Apaydin et al., 2000; Agarwal et al., 2004; Matuck et al., 2004; Vikrant et al., 2005; Naqvi et al., 2006; Naqvi et al., 2010; Kim et al., 2011; de Lemos et al., 2013; Kim et al., 2013; Kim et al., 2015; Kim et al., 2020; Dodani et al., 2021). The basic characteristics of these 13 studies are shown in Table 1. Four studies were from the Republic of Korea (Kim et al., 2011; Kim et al., 2013; Kim et al., 2015; Kim et al., 2020), three each from India (John et al., 1994; Agarwal et al., 2004; Vikrant et al., 2005) and Pakistan (Naqvi et al., 2006; Naqvi et al., 2010; Dodani et al., 2021), two from Brazil (Matuck et al., 2004; de Lemos et al., 2013), and one study was from Turkey (Apaydin et al., 2000). The sample size of the studies ranged from 85 (Vikrant et al., 2005) to 1,760 (Dodani et al., 2021). Nine studies reported mean follow-up periods ranging from 12 to 252 months, while the other studies did not clarify the follow-up time. Of these nine studies, six were randomized controlled trials (RCTs) (John et al., 1994; Agarwal et al., 2004; Vikrant et al., 2005; Naqvi et al., 2006; Naqvi et al., 2010; Kim et al., 2015), four were cohort studies (Kim et al., 2011; de Lemos et al., 2013; Kim et al., 2020; Dodani et al., 2021), two were retrospective studies (Apaydin et al., 2000; Matuck et al., 2004), and one was a prospective study (Kim et al., 2013). Most of the included studies reported an INH prevention time of more than 9 months, the longest being 12 months. The quality of the 13 eligible studies assessed by Cochrane’s collaboration tool is shown in Figure 2. A total of six studies were RCTs, and only three studies detailed the process of random sequence generation. The eligible studies had a moderate risk of bias.

**TABLE 1:** Basic characteristics of included studies.

Author (year)	Country	Simple size	Design	Prophylaxis time (months)	Follow-up (months)	Active TB	All-cause mortality	Hepatotoxicity	AR
Dodani et al. (2021)	Pakistan	910/850	Retrospective cohort	12	58.8	46/130	20/NR	18/NR	7/50
Kim et al. (2020)	Republic of Korea	105/1,045	Retrospective cohort	9	31.5	0/12	1/20	1/2	4/55
Kim et al. (2015)	Republic of Korea	131/132	RCT	9	21.7	0/3	3/2	5/NR	20/18
Kim et al. (2013)	Republic of Korea	10/87	Prospective	NR	24.6	0/1	NR	NR	NR
de Lemos et al. (2013)	Brazil	274/261	Retrospective cohort	6	59	2/9	42/48	NR	112/68
Kim et al. (2011)	Republic of Korea	40/272	Longitudinal cohort	9	14.5	0/4	0/2	NR	4/18
Naqvi et al. (2010)	Pakistan	181/207	RCT	12	NR	1/16	0/0	1/0	19/26
Naqvi et al. (2006)	Pakistan	187/215	RCT	12	24	1/10	NR	0/0	19/22
Vikrant et al. (2005)	India	42/43	RCT	12	NR	9/18	12/10	27/18	NR
Matuck et al. (2004)	Brazil	30/982	Retrospective	NR	252	1/44	NA/14	0/NR	0/NR
Agarwal et al. (2004)	India	27/58	RCT	12	12	3/15	1/0	1/0	NR
Apaydin et al. (2000)	Turkey	51/223	Retrospective	6	NR	3/13	NR	NR	1/NR
John et al. (1994)	India	92/92	RCT	12	NR	3/4	NR	32/33	NR

TB, tuberculosis; AR, acute rejection; RCT, randomized controlled trial; NR, not reported.

### Primary outcomes

**FIGURE 3:**
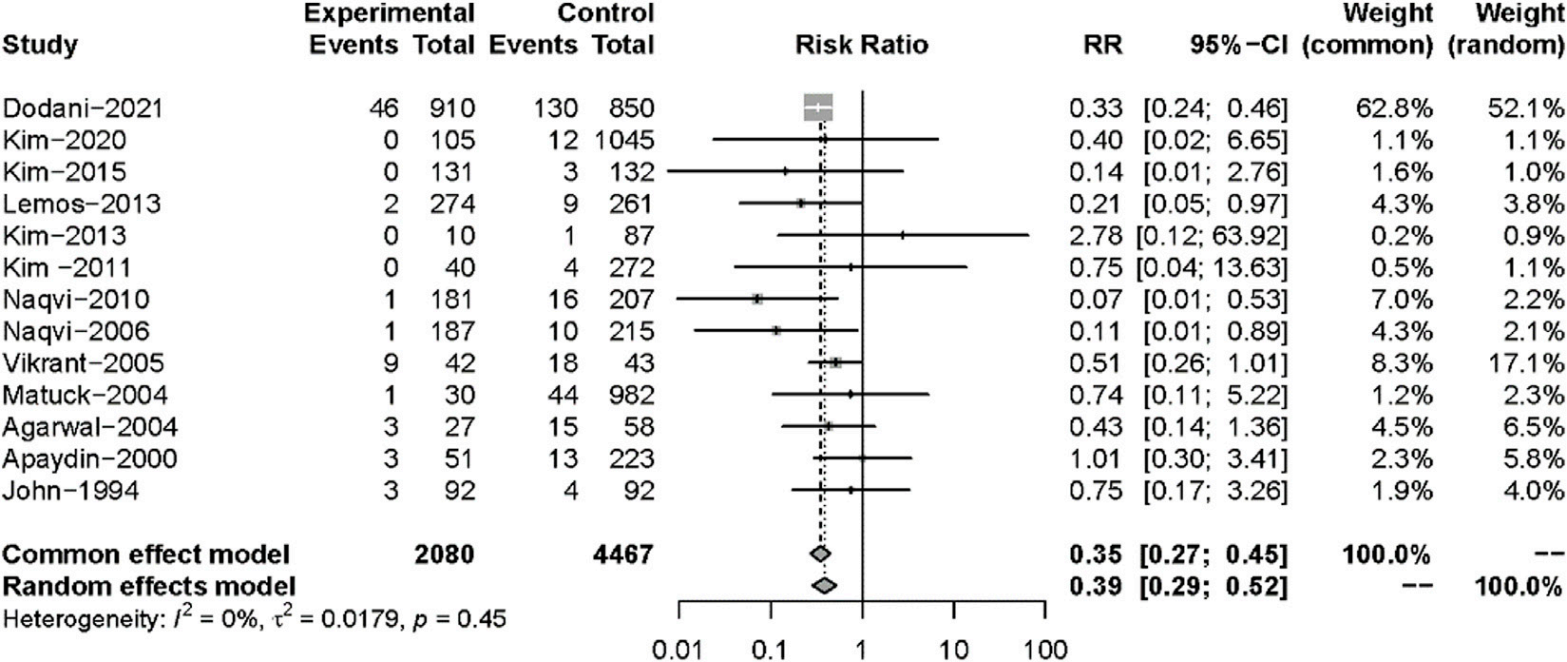
Forest plot for outcomes of active TB comparing isoniazid *versus* no treatment.

**FIGURE 4:**
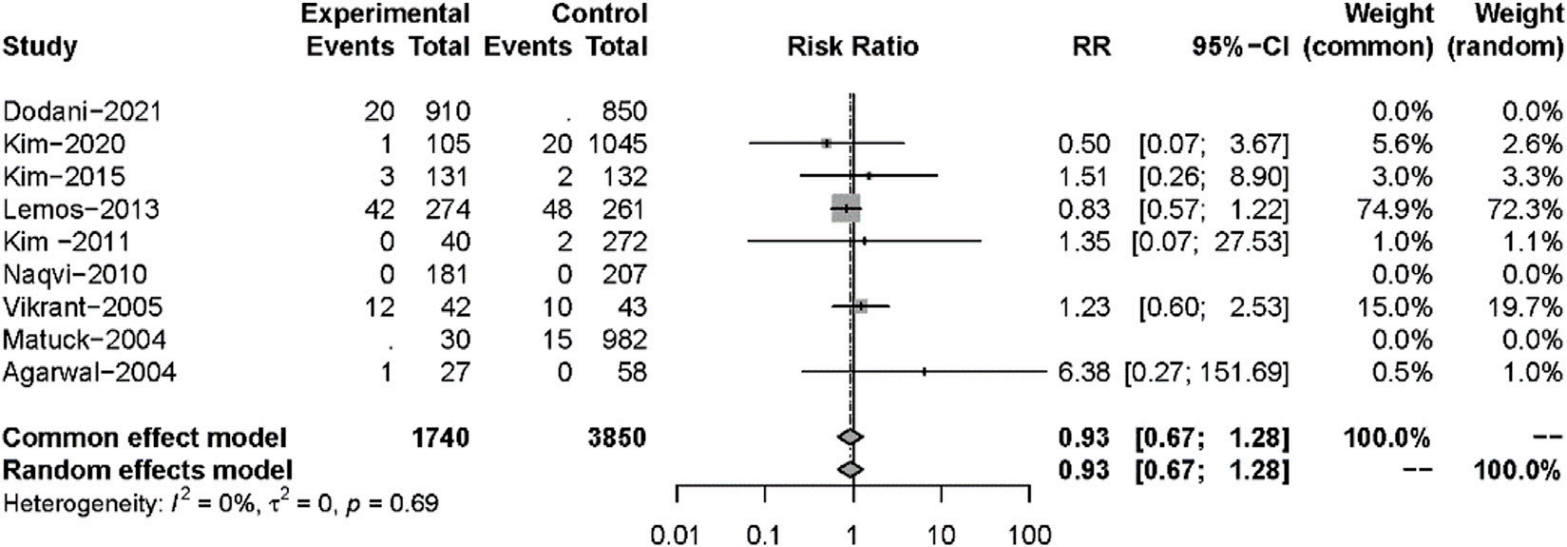
Forest plot for outcomes of all-cause mortality comparing isoniazid *versus* no treatment.

All 13 studies reported TB infections. The INH prophylaxis group had a lower risk of infection than the non-INH prophylaxis group (RR: 0.35, 95%CI 0.27–0.45, *p* < 0.01) (Figure 3), with no heterogeneity among these studies (I^2^ = 0%, *p* = 0.45). Nine studies reported the results of all-cause mortality (Figure 4), and no significant difference was found between the two groups (RR: 0.93, 95%CI 0.67–1.28, *p* = 0.64), with no heterogeneity among the nine studies (I^2^ = 0%, *p* = 0.69).

### Secondary endpoints

**FIGURE 5:**
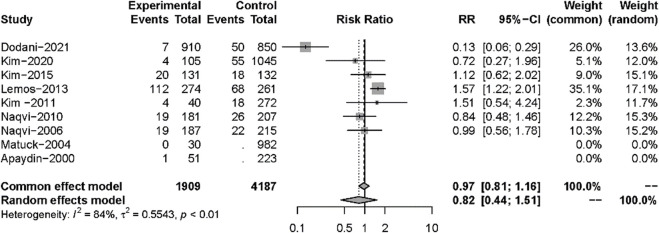
Forest plot for outcomes of acute rejection comparing isoniazid *versus* no treatment.

**FIGURE 6:**
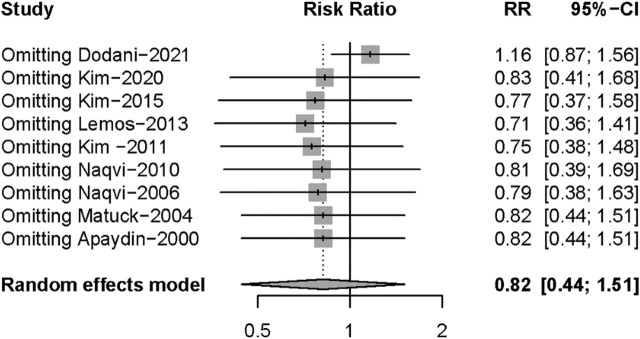
Sensitivity analyses for acute rejection.

**FIGURE 7:**
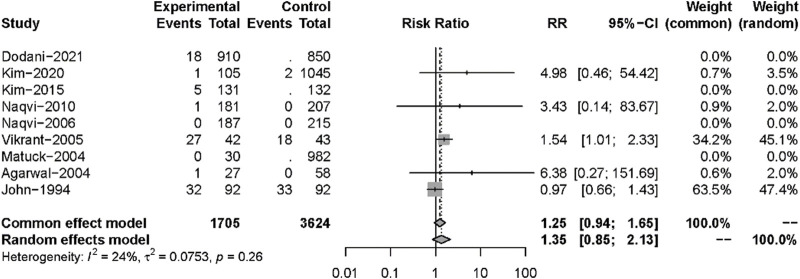
Forest plot for outcomes of hepatotoxicity comparing isoniazid *versus* no treatment.

Nine studies described posttransplant ARs. Despite there being no significant difference in both groups (RR: 0.82, 95%CI 0.44–1.51, *p* = 0.52), significant heterogeneity was found in these studies (I^2^ = 84%; *p* < 0.01) (Figure 5). Additional sensitivity analyses to evaluate the variance found that the work of Dodani et al. (2021)might be responsible for the source of heterogeneity (Figure 6). We failed to find any difference in the subgroup analysis of ARs in Supplementary Figure S1, and we found no difference in these studies by subgroup analysis of intervention and observational studies (Supplementary Figure S2). There was also no difference in hepatotoxicity between the two groups (RR: 1.25, 95%CI 0.94–1.65, *p* = 0.12), and no significant heterogeneity was found among these studies (I^2^ = 24%, *p* = 0.26) (Figure 7).

## Discussion

This meta-analysis (involving 6,547 KTRs) found that there was a significantly lower risk of active TB infection among KTRs who received INH prophylaxis. However, there were no significant differences between the groups on mortality and hepatotoxicity. Furthermore, ARs were not significantly different between the two groups, and the corresponding sensitive analysis showed no statistically significant difference. Thus, the application of INH is a safe and effective strategy for preventing TB infection after KT.

TB is one of the most common infections with negative impact post-transplantation (Karuthu and Blumberg, 2012). As KT is correlated with an immunosuppression status, the morbidity of active TB is obviously higher in KT recipients than in the general population (Torre-Cisneros et al., 2009; Kwon et al., 2021). It is reported that mortality can reach 60% (Lezaic et al., 2001), and graft rejection can reach 55.6% (el-Agroudy et al., 2003) in KT recipients with TB. Apaydin et al. (2000) found no statistical difference in the development of active TB after kidney transplantation in the INH treatment group compared with non-INH. In contrast, Dodani et al. (2021) conducted a study on primary INH prophylaxis in renal transplant recipients and found that the incidence of active TB decreased in the first two years. In addition, a systematic review of renal transplant recipients with TB suggested that INH prophylaxis was less likely to develop active TB compared with those who did not receive this treatment (Adamu et al., 2014). However, many transplant centers do not routinely use INH prophylaxis because of an increased risk of liver toxicity after INH treatment (Antony et al., 1997). Our study showed that INH prophylaxis can be beneficial in reducing the risk of TB infection and has no difference in side effects. Vikrant et al. (2005) explained that viral hepatitis was very common during dialysis, and that hepatotoxicity caused by viral hepatitis was sometimes difficult to distinguish from hepatotoxicity caused by INH. Meanwhile, the American Thoracic Society recommended that INH should be stopped only when liver enzyme levels increased three to five times in symptomatic patients (Van Stralen et al., 2013). Therefore, we still need to routinely monitor liver function based on these findings.

Our study had several limitations. First, there were differences in study design (six RCTs, four cohort studies, two retrospective studies, and one prospective study), leading to inherent bias. Second, some studies provided incomplete data, preventing a comprehensive review. Third, the diagnosis of latent TB was not the gold standard. Tuberculin skin test (TST) had limited sensitivity in renal failure patients, and the validity of interferon-gamma release assay was still uncertain in the immunocompromised population. However, INH prophylaxis therapy has been empirically initiated in some studies. Fourth, the outcomes of these studies may be influenced by many potential confounders. Fifth, the follow-up time in this study differed greatly from 12 months to 252 months. Finally, the prophylaxis period was not uniform, from 6 to 12 months, which may require new clinical studies for different prevention times to confirm its safety and effectiveness.

In conclusion, this meta-analysis revealed that INH prophylaxis could significantly reduce the risk of TB development in KTRs. Nevertheless, the available evidence is not robust and a large multicenter randomized trial is needed to evaluate the efficacy and safety of INH prophylaxis in KTRs (Bishai and Chaisson, 1997, Muñoz et al., 2005, Riella, 2018, Samavat et al., 2021, Aguado et al., 2009).

## Data Availability

The original contributions presented in the study are included in the article/Supplementary Material; further inquiries can be directed to the corresponding author.

## Abbreviations

INH, isoniazid; KTRs, kidney transplant recipients; RR, risk ratios; AR, acute rejection; KT, kidney transplantation; ESKD, end-stage kidney disease; TB, tuberculosis; SOT, solid organ transplantation; LTBI, latent tuberculosis infection; MD, mean difference; CI, confidence interval; RCTs, randomized controlled trials; CYP3A4, cytochrome P450; TST, tuberculin skin test.
