# Supplementary material for: Chemoprophylaxis for the prevention of tuberculosis in kidney transplant recipients: A systematic review and meta-analysis
Source: Front Pharmacol. 2023 Mar 16;14:1022579. doi: 10.3389/fphar.2023.1022579 (PMC10060851; doi:10.3389/fphar.2023.1022579)
Supplement: Supplementary file 2 [file Table2.pdf]

Table S2. The searched items in the database of Web of science, PubMed and Scopus.

Database	Web of science	Pubmed	Scopus
Logical combination of relevant words	renal transplant OR kidney transplant OR renal allograft OR kidney allograft (theme) AND tuberculosis OR tuberculoses (theme)	"renal transplant"[Title/Abstract] OR "kidney transplant"[Title/Abstract] OR "renal allograft"[Title/Abstract] OR "kidney allograft"[Title/Abstract]) AND AND ("tuberculosis"[Title/Abstract] OR "tuberculoses"[Title/Abstract])	(TITLE-ABS-KEY (renal AND transplant OR kidney AND transplant] OR renal AND allograft OR kidney AND allograft) AND TITLE-ABS-KEY (tuberculosis OR tuberculoses))
Range of retrieval time	1966/06/10 to 2022/06/10	1966/06/10 to 2022/06/10	1966/06/10 to 2022/06/10
Records	1120	490	208
