# Supplementary material for: Chemoprophylaxis for the prevention of tuberculosis in kidney transplant recipients: A systematic review and meta-analysis
Source: Front Pharmacol. 2023 Mar 16;14:1022579. doi: 10.3389/fphar.2023.1022579 (PMC10060851; doi:10.3389/fphar.2023.1022579)
Supplement: Supplementary file 3 [file Image2.pdf]

(A)

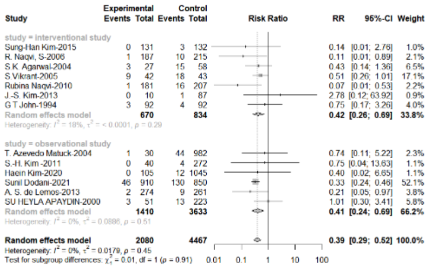

(B)

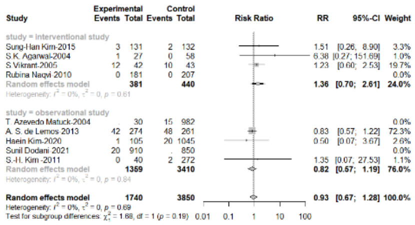

(C)

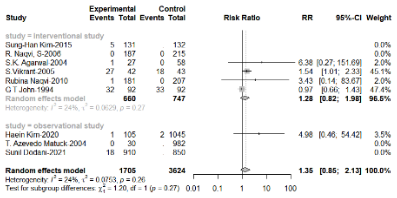

(D)

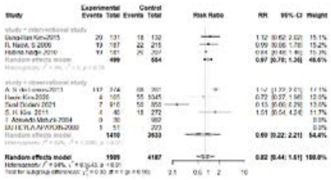

Figure S2. The subgroup analysis between observational and interventional studies. (A). The active TB infections after transplantation, (B) The all-cause mortality, (C) The hepatotoxicity, (D) The ARs.
