# Supplementary material for: Chemoprophylaxis for the prevention of tuberculosis in kidney transplant recipients: A systematic review and meta-analysis
Source: Front Pharmacol. 2023 Mar 16;14:1022579. doi: 10.3389/fphar.2023.1022579 (PMC10060851; doi:10.3389/fphar.2023.1022579)
Supplement: Supplementary file 4 [file Image1.pdf]

(A)

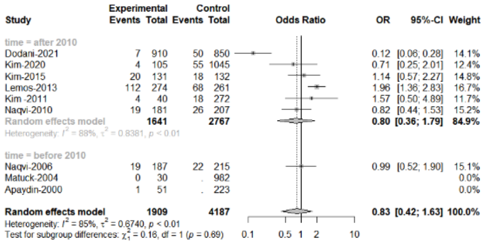

(B)

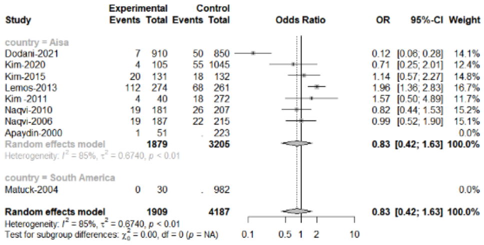

(C)

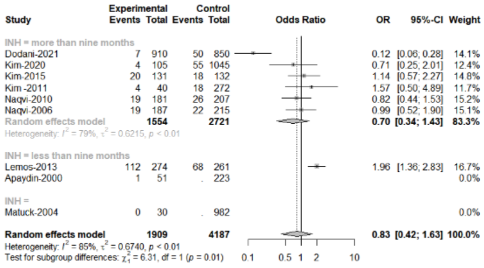

(D)

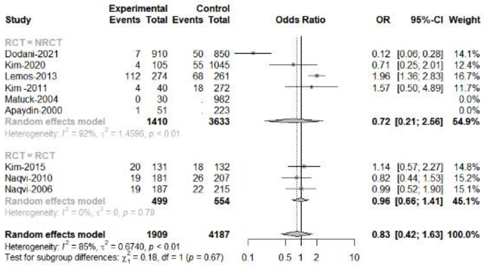

Figure S1. The subgroup analysis of the acute rejections in both groups. (A). The time of study publication, (B) The area of study, (C) The time of INH prophylaxis, (D) The included study type.
